# The potential protective role of vitamin D and calcium supplements in reducing cardiovascular disease risk among elderly patients with osteopenia

**DOI:** 10.1007/s11845-024-03709-2

**Published:** 2024-05-14

**Authors:** Ramada R. Khasawneh, Hana S. Al-Soudi, Ejlal Abu-El-Rub, Ayman Alzu’bi, Raed M. Al-Zoubi

**Affiliations:** 1 Department of Basic Medical Sciences, Faculty of Medicine, Yarmouk University, Irbid, 211-63 Jordan; 2 Nuclear Medicine, King Hussein Medical Center, Royal Medical Services, Amman, Jordan; 3 Surgical Research Section, Department of Surgery, Hamad Medical Corporation & Men’s Health, Doha, Qatar; 4 Department of Biomedical Sciences, QU-Health, College of Health Sciences, Qatar University, Doha, 2713 Qatar; 5 Department of Chemistry, Jordan University of Science and Technology, P.O. Box 3030, Irbid, 22110 Jordan; 6 Surgical Research Section, Department of Surgery, Hamad Medical Corporation, Doha, Qatar

**Keywords:** Bone mineral density, Calcium, Cardiovascular disease, Dax scan, Vitamin D

## Abstract

**Background:**

Cardiovascular disease and low bone mineral density are major health problems in the elderly. These two conditions are considered independent of each other and age-related diseases. The aim of this study is to investigate the association between low bone mineral density (BMD) and cardiovascular disease (CVD) incidents, and the effect of vitamin D and calcium supplement on the incidence of CVD in patients with low BMD.

**Methods:**

A total of 1047 patients (597 females/450 males) with the age of 65 years and more were diagnosed with osteopenia for 13 years or more. The study also included 220 patients (107 females/113 males) with osteopenia who already took calcium and vitamin D continually since their diagnosis. BMD was measured by dual-energy X-ray absorptiometry. The incidence of any cardiovascular diseases in the study patients and the presence of corresponding risk factors were collected and analyzed.

**Results:**

In both elderly Arab females and males, there was an association between total hip and femoral neck BMD and the possibility to have CVD. On the other hand, the results showed that patients who use calcium and vitamin D supplements showed a significant reduction in the incidence of CVD comparing to the non-treated patients.

**Conclusion:**

Low total hip and femoral neck BMD were associated with a higher chance to have CVD incidents in both elderly Arab males and females; moreover, calcium and vitamin D supplements have a possible protective role in reducing cardiovascular disease in elderly patients with osteopenia.

## Introduction

Cardiovascular diseases (CVD) and low bone mineral density (BMD) are serious health problems that hit many countries and cause significant morbidity and mortality. Both CVD and low BMD often occur concurrently and are associated with shared risk factors such as age, smoking, low physical activity, and hypertension [1]. Due to the life-threatening consequences of both diseases, recurrent prolonged hospitalizations may be needed, which impose heavy burden on the healthcare system of many countries.

Although CVD and low BMD are thought initially as unrelated diseases, several studies investigated the possible relationship between the two conditions. Many elderly patients who were diagnosed to have heart disease found to have low BMD as one of the contributing risk factors [2–4]. Some studies suggested a noticeable increase in acute myocardial infarction incidents in elderly people who have experienced fractures [5, 6].

Epidemiological studies have found that low BMD predisposes postmenopausal female patients to coronary heart disease and stroke [7]. Moreover, a study conducted by the American Heart Association revealed that 38% of people with osteopenia also have atrial fibrillation [8].

Research suggests a noteworthy link between BMD and an elevated risk of CVD; several studies indicated that low BMD can cause CVD by increasing the tendency to have vascular calcification that leads to narrowing and clogging the lumen of coronary vessels [9, 10]. The increase in the risk of vascular calcification in patients with low BMD contributes to the stiffening of coronary vessels and the development of atherosclerosis [10, 11]. Moreover, fractures resulting from reduced bone density can serve as a warning sign for potential cardiovascular issues mediated by enkindling massive inflammation that can affect the normal heart function [12].

The available literature regarding the relation between low BMD and CVD still has some contradictions regarding the possible relation between the two conditions. Relatively, few studies have addressed the relation between low BMD and the CVD. Diverse ethnicities may influence the relation between low BMD and CVD. Some studies investigated the impact of race on the relationship between BMD and CVD by comparing blacks and whites [13, 14]. The studies found that lower total hip BMD was associated with higher heart failure (HF) risk in white men but lower risk in black men. According to our knowledge, there is no study that described the relationship between low BMD and CVD in the Arab population.

Calcium and vitamin D play an important role in mineral homeostasis and the maintenance of skeletal health. Calcium and vitamin D supplements have been widely used for fracture prevention in elderly populations. Many trials have studied the effectiveness and cardiovascular safety of calcium and vitamin D supplementation, with disparate results [15, 16]. In this study, we evaluated the effect of calcium and vitamin D supplements on decreasing the risk of CVD in elderly Arabian patients with low bone mineral density using dual-energy X-ray absorptiometry (DXA). The findings of this study can be used for future comparative studies by providing an evidence that can confirm or disprove the association between low BMD and CVD.

## Materials and methods

### Study sample

This retrospective study was approved by the institutional research board at Jordan University of Science and Technology (IRB # 5/135/2020).

The study participants were included from King Abdullah University Hospital. The subjects were 65 years and more who were diagnosed with osteopenia for 13 years or more (from 2011 until 2020) and have complete filed datasets.

Data extraction from the database was performed independently by two researchers. Any disagreement between the two recruited researchers was solved by a third researcher, who also was anonymous to the other two researchers. A total of 1047 (597 females/450 males) subjects were included in the study. Patients who had thyroid gland, parathyroid gland, and adrenal gland disorders were excluded. Patients who had a history of significant bone injury, liver failure, chronic diseases of gastrointestinal tract, cancer or life-threatening illness, diabetes mellitus, and smoking were also excluded from the study. The baseline characteristics of the study group, stratified by gender, are summarized in Table 1.

**Table 1:** Clinical characteristics of the study cohort stratified by gender

Characteristics	Females (*n* = 597)	Males (*n* = 450)	*P*-value
Age (years)	76.2 ± 6.1	77.8 ± 5.9	0.2
Body mass index (kg/m^2^)	26.6 ± 4.6	25.3 ± 3.4	< 0.05
Systolic blood pressure (mm Hg)	134 ± 22	131 ± 18	< 0.05
Low-density lipoprotein (mg/dL)	132 ± 35	125 ± 28	< 0.05
High-density lipoprotein (mg/dL)	55 ± 12	46 ± 9	< 0.05
Triglycerides (mg/dL)	125 (80–171)	115 (73–142)	< 0.05
Lipid-lowering medication, number (%)	134 (22.4%)	121 (26.8%)	0.493
Aspirin, No. (%)	228 (38.1%)	184 (40.8%)	0.329
Prevalent coronary heart disease, No. (%)	91 (15.2%)	127 (28.2%)	< 0.05
Prevalent stroke/transient ischemic attack, No. (%)	51 (8.5%)	41 (9.1%)	0.493
Prevalent peripheral arterial disease, No. (%)	62 (10.3%)	46 (10.2%)	0.641
Prevalent atrial fibrillation, No. (%)	31 (5.19%)	29 (6.4%)	0.519
Serum creatinine (mg/dL)	0.9 ± 0.3	1.1 ± 0.2	0.172
Estimated glomerular filtration rate (cystatin-based) (mL/min per 1.73 m^2^)	84 ± 18	81 ± 15	0.227
C-reactive protein (mg/L)	2.4 (1.2–5.6)	1.9 (1–4.2)	
Total hip BMD (g/cm^2^)	0.7 ± 0.1	0.9 ± 0.1	< 0.001
Femoral neck BMD (g/cm^2^)	0.6 ± 0.1	0.8 ± 0.1	< 0.001
Total hip, WHO categories, No. (%)			< 0.001
Normal	223 (37.3%)	200 (44.4%)	
Osteoporosis	374 (62.6%)	250 (55.6%)	
Femoral neck, WHO categories, No. (%)			< 0.001
Normal	268(44.8%)	207 (46%)	
Osteoporosis	329 (55.1%)	243 (54%)	

Data for continuous variables are presented as mean ± SD

*BMD* bone mineral density, *WHO* World Health Organization

Patients with osteopenia used a bisphosphonate or denosumab as a treatment course. The number of osteopenia patients who were on calcium and vitamin D3 medications in addition to bisphosphonate or denosumab was 220 patients, and the subjects were 65 years and more and diagnosed with osteopenia for 10 years or more. The records of those patients showed that they were on calcium (1000–1200 mg daily) and vitamin D3 (600–800 IU daily) since their diagnosis. Those patients, who underwent treatment, were followed up by performing regular dual-energy X-ray absorptiometry (DXA) scans. Among the 220 patients, there were 107 females (48 females were diagnosed with femoral neck osteopenia, 59 females with total hip osteopenia) and 113 males (52 males were diagnosed with femoral neck osteopenia, 61 males with total hip osteopenia).

### Measurement of bone mineral density

Dual-energy X-ray absorptiometry (DXA) scans were performed (Hologic 4500A, version 9.03; Hologic, Inc., Waltham, MA, USA) to measure bone mineral density (BMD), using the array beam mode. Scans were read blindly at the King Abdullah University Hospital using Hologic software version 9.03. The primary measures of interest were BMD of the total hip and BMD of the femoral neck. DXA scan protocols that were used for all subjects were identical.

For the DXA scan results, the World Health Organization classification chart was used for osteopenia using a T-score. The T-scores were categorized as follows: (i) normal, when the T-score is − 0.99 or above; (ii) osteopenia, the T-score between − 1 and − 2.5; (iii) osteoporosis, the T-score below − 2.5.

### The incidence of cardiovascular diseases in osteopenia patients

The recruited expert panel reviewed all relevant CVD data in the files of the patients included in the study. The history, physical examination, report of chest radiography, and medication usage were assessed. In this study, the incidence of cardiovascular disease was defined as the initiation of one or more of the heart conditions after being diagnosed with osteopenia. The CVDs included in this study are coronary heart disease, stroke/transient ischemic, peripheral arterial disease, and atrial fibrillation. For atrial fibrillation confirmation, the experts combined the annual ECGs performed for the recruited patients upon hospitalization. Hospital records for the recruited patients were collected, abstracted, and reviewed by the attending cardiologist.

### Assessment and definition of baseline variables

We obtained the following baseline variables: body weight, height, systolic blood pressure, smoking, glucose level, C-reactive protein, and treatment with antihypertensive medication from the patient’s medical records.

Smoking increases mortality from all causes and has a crucial role in atherosclerotic cardiovascular disease [17], so we excluded the smoker patients from this study. Moreover, patients with diabetes mellitus who were prescribed hypoglycemic medications were excluded as well.

Body mass index (BMI) was calculated as weight divided by height squared (kg/m^2^). Patients with a BMI above 30 are excluded from the study to minimize the influence of obesity-related confounding variables, which is known as a risk factor for CVD and bone disorders. Moreover, patients using antihypertensive medications were excluded from the study populations. Cystatin C was used to calculate the estimated glomerular filtration rate (eGFR) using standard methods.

### Statistical analysis

Baseline characteristics and BMD measures of groups with or without incident cardiovascular disease in each sex group were performed using *t*-tests for unequal variances and the chi-square test for categorical and continuous variables. Total hip and neck BMD were evaluated both as continuous and categorical variables.

Post hoc pair-wise tests were planned to ascertain any group differences. Statistical significance was tested at the level of *P* = 0.05. The data are presented as mean ± standard error of the mean (SEM).

## Results

### The association between the baseline variables and BMD

This study included 1047 subjects with a mean age of 77 ± 6 years. Of these, 57% were women and 43% were men. Females in this study were younger in comparison to males and had more prevalent use of lipid-lowering drugs and aspirin and had higher eGFR levels and triglycerides (Table 1).

Results describing the association between baseline variables and total hip BMD and femoral neck BMD are summarized in Tables 2 and 3.

**Table 2:** The association of continuous total hip BMD with baseline characteristics of the study cohort

Characteristics	Total hip BMD							
	Females (*n* = 597)				Males (*n* = 450)			
	No.	Correlation coefficient	Mean ± SD	*P*-value	No.	Correlation coefficient	Mean ± SD	*P*-value
Age (year)	597	− 0.32		< 0.05	450	− 0.21		< 0.05
Body mass index (kg/m^2^)	597	0.52		< 0.05	450	0.36		< 0.05
Systolic blood pressure (mm Hg)	597	0.03		0.505	450	0.03		0.510
Low-density lipoprotein (mg/dL)	523	0.001		0.982	425	0.03		0.524
High-density lipoprotein (mg/dL)	596	˗0.05		0.149	450	0.01		0.892
Triglycerides (mg/dL)	597	0.12		0.004	450	0.09		0.04
Lipid-lowering medication								
Yes	134		0.75 ± 0.14	0.313	121		0.95 ± 0.15	0.332
No	463		0.77 ± 0.15		329		0.92 ± 0.18	
Aspirin								
Yes	228		0.75 ± 0.15	0.457	184		0.95 ± 0.16	0.742
No	369		0.75 ± 0.13		266		0.95 ± 0.16	
Prevalent coronary heart disease								
Yes	91		0.75 ± 0.14	0.771	127		0.95 ± 0.16	0.492
No	506		0.75 ± 0.15		323		0.95 ± 0.18	
Prevalent stroke/transient ischemic attack								
Yes	51		0.75 ± 0.15	0.928	41		0.95 ± 0.18	0.255
No	546		0.75 ± 0.14		409		0.95 ± 0.16	
Prevalent peripheral arterial disease								
Yes	508		0.75 ± 0.14	0.937	367		0.96 ± 0.19	0.167
No	89		0.74 ± 0.15		83		0.91 ± 0.16	
Prevalent atrial fibrillation								
Yes	31		0.69 ± 14	0.011	29		0.95 ± 0.18	0.968
No	566		0.75 ± 15		421		0.95 ± 0.16	
Creatinine (mg/dL)	595	0.07		0.062	426	− 0.01		0.914
Estimated glomerular filtration rate (cystatin-based) (mL/min per 1.73 m)	586	0.06		0.086	439	0.09		0.033
C-reactive protein (mg/L)	595	0.11		0.004	450	0.02		0.734

**Table 3:** The association of continuous femoral neck BMD with baseline characteristics of the study cohort

Characteristics	Total hip BMD							
	Females (*n* = 597)				Males (*n* = 450)			
	No.	Correlation coefficient	Mean ± SD	*P*-value	No.	Correlation coefficient	Mean ± SD	*P*-value
Age (year)	195	− 0.30		< 0.05	152	− 0.21		< 0.05
Body mass index (kg/m^2^)	195	0.49		< 0.05	152	0.32		< 0.05
Systolic blood pressure (mm Hg)	195	0.02		0.505	152	0.02		0.510
Low-density lipoprotein (mg/dL)	523	˗0.01		0.8	425	0.02		0.62
High-density lipoprotein (mg/dL)	596	˗0.03		0.42	450	0.01		0.812
Triglycerides (mg/dL)	597	0.06		0.06	450	0.04		0.831
Lipid-lowering medication								
Yes	134		0.65 ± 0.12	0.77	121		0.78 ± 0.17	0.458
No	463		0.65 ± 0.15		329		0.77 ± 0.15	
Aspirin								
Yes	65		0.64 ± 0.15	0.457	184		0.78 ± 0.16	0.322
No	130		0.65 ± 0.12		266		0.79 ± 0.16	
Prevalent coronary heart disease								
Yes	91		0.64 ± 0.12	0.921	126		0.78 ± 0.16	0.672
No	506		0.64 ± 0.15		324		0.78 ± 0.17	
Prevalent stroke/transient ischemic attack								
Yes	22		0.64 ± 0.12	0.827	41		0.78 ± 0.18	0.255
No	575		0.64 ± 0.14		409		0.76 ± 0.16	
Prevalent peripheral arterial disease								
Yes	508		0.65 ± 0.14	0.826	367		0.76 ± 0.18	0.651
No	89		0.65 ± 0.12		83		0.77 ± 0.15	
Prevalent atrial fibrillation								
Yes	31		0.62 ± 12	0.013	34		0.77 ± 0.17	0.446
No	566		0.65 ± 13		416		0.74 ± 0.16	
Creatinine (mg/dL)	595	0.07		0.062	426	0.002		0.914
Estimated glomerular filtration rate (cystatin-based) (mL/min per 1.73 m)	586	0.06		0.115	439	0.08		0.123
C-reactive protein (mg/L)	595	0.14		0.002	450	0.04		0.371

In both sexes, all BMD measurements were negatively correlated with age. However, the BMD measurements showed a statistically significant association with a high triglyceride level. The incidence of atrial fibrillation was more frequent in patients with lower total hip BMD. Males with high total hip BMD were found to have a higher eGFR level.

### The incidence of cardiovascular disease

The filed database of the patients recruited in the current study incorporated all the medical information, follow-up visits, regular medical examinations, images, and regular DXA scans after being diagnosed with osteopenia.

The records revealed 478 incidents of cardiovascular disease (CVD), with 235 (49.2%) occurring in females and 243 (50.8%) occurring in males. The number and incidence rates of CVD in relation to BMD categories were summarized in Table 4. The highest event rates occurred in females with osteopenia of the total hip. The analysis showed that the incidence of CVD significantly increased in patients with total hip osteopenia comparing to individuals with normal BMD. Moreover, the incidence of CVD significantly increased in patients with femoral neck osteopenia comparing to individuals with normal BMD. The results also showed that patient gender did not affect this CVD incidence related to BMD abnormalities.

**Table 4:** CVD events with normal BMD and osteopenia stratified by sex

**CVD event**	**Female**	**Male**
Total hip: No. (incidence rate)		
Normal BMD	55 (24.6%)	31 (15.5%)
Osteopenia	180 (48.1%)*	55 (22%)*
Femoral neck (incidence rate)		
Normal BMD	33 (12.3%)	31 (14.9%)
Osteopenia	98 (29.7%)*	51 (20.9%)*

*BMD* bone mineral density, *CVD* cardiovascular disease

*P < 0.05 (*t*-test). The significant increase was between normal and osteopenia

Based on the above findings, there was an obvious association between BMD and CVD incidents in both males and females.

### Correlation of low level of daily vitamin D and calcium with the risk of cardiovascular diseases

There is no cure for osteopenia. The goal of osteopenia treatment is to prevent its progression to osteoporosis which increases the fracture risk. The main treatment for osteopenia is bisphosphonate or denosumab with or without calcium and vitamin D3 supplements.

**Fig. 1:**
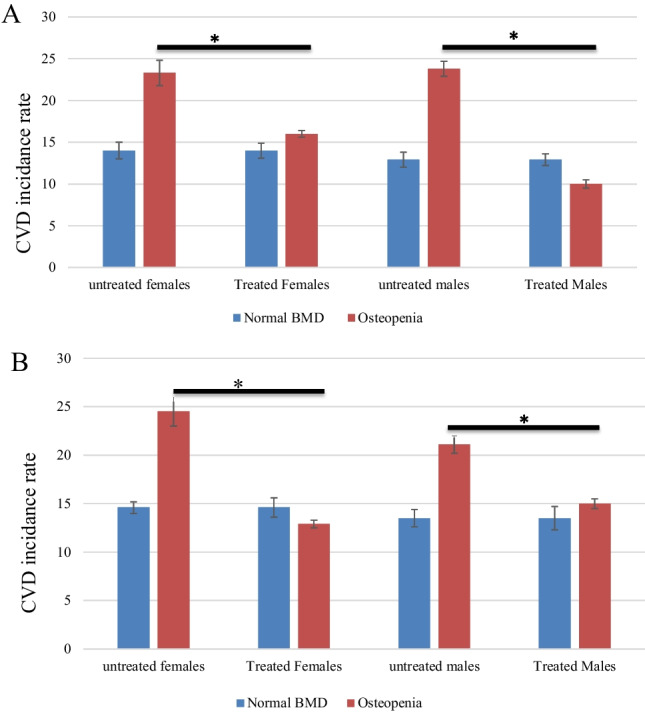
Correlation of low level of daily vitamin D and calcium with the risk of CVD events. **A** The comparison of the incidence of CVD between patients treated with calcium and vitamin D and the non-treated patients with normal BMD. Total hip osteopenia, a significant increase in the incidence of CVD, was in the non-treated group compared to the treated group. **B** The comparison of the incidence of CVD between patients treated with calcium and vitamin D and the non-treated patients with normal BMD. Femoral neck osteopenia, again, a significant increase in the incidence of CVD, was in the non-treated group compared to the treated group. Each column represents the percentage of incidence of CVD ± standard error of the mean (SEM). *P < 0.05 (*t*-test)

In this study, 220 patients (107 females, 113 males) were diagnosed with osteopenia and were on daily calcium and vitamin D3 supplements since their diagnosis. The data showed that the treated patients that used calcium and vitamin D3 supplements had a significant reduction in the incidence of CVD comparing to the treated patients that did not use calcium and vitamin D3 supplements. Moreover, the incidence of CVD in the treated patients was similar to the healthy individuals (Fig. 1).

## Discussion

Recent studies have shed light on a potential relationship between low BMD and CVD, suggesting that the two conditions may be interconnected [1, 3, 18]. Low BMD and heart diseases may share common risk factors such as aging, sedentary lifestyle, poor diet, and certain medical conditions. Moreover, evidence has suggested that individuals with low BMD are susceptible to CVD [19, 20]. In this study, we exclude the major risk factors that are commonly associated with CVD which made this study to focus more readily on the association between BMD and CVD.

In this study, we evaluated the possible association between low BMD and CVD using a cohort of the Arab population who underwent a DAX scan. We found that BMD measurements can be used as an indicator for future CVD in both sexes, particularly HF. The subjects selected for this study did not have a history of CVD before being diagnosed with osteopenia, and they had CVD after a while of having osteopenia onset.

The outcomes of this study revealed a possible relation between low BMD and the incidence of CVD, and it is considered a non-gender-related correlation. An earlier observation by Farhat et al. studied the incidence of CVD and reported an inverse association between BMD and CVD in white males [21] but not in white females. The association between low BMD and CVD in males was also reported by Laroche et al. who recruited 18 males with asymmetrical symptomatic peripheral arterial disease. Laroche et al. showed that the BMD was significantly lower in the affected leg in comparison to the unaffected leg [22]. The possible explanation for the relationship between BMD and the incidence of HF in males can be due to the intense inflammation and high cytokine levels such as IL-6, TNF-α, and oxLDL that accompany the osteopenia in males [23]. As patient age increases, the decrease in the level of endogenous sex steroid hormones leads to an imbalance in the calciferol endocrine system [24, 25]. Patients with osteopenia are found to have sometimes a high level of PTH as a part of the pathological mechanism that causes bone thinning. Elevated PTH in osteopenia have been found to increase the incidence of HF particularly in males [26]. These suggested mechanisms are plausible, and future investigations are needed to elucidate the validity of these explanations.

In females, we observed an association between BMD and incident CVD, and this also was in consistent with Fohtung et al., who found an insignificant impact of low BMD on triggering HF incidents in females [13]. All women were over the age of 60 and suffering from menopause, which being suggested as a dominant risk factor for osteopenia.

Many patients with low BMD suffer from calcium and vitamin D deficiency. Calcium and vitamin D supplementations were effective in reducing osteoporotic fractures. Studies showed a reduction in the osteoporotic fractures between 25 and 70% with a daily intake of approximately 1000 mg/day of elemental calcium [27, 28]. Another study showed that calcium repletion with marginal vitamin D levels in study subjects resulted in bone sparing [27]. Therefore, optimal intake of vitamin D is influenced by calcium intake [27]. Vitamin D has been extensively studied regarding its impact on fracture risk reduction. In fact, vitamin D deficiency has been associated with a greater incidence of hip fracture in many populations [29]. Vitamin D is required for calcium absorption, and its deficiency increases the rates of bone loss which can increase the risk of fracture [28]. Studies also showed that concomitant supplementation of vitamin D and calcium may decrease fat deposition and reduce the risk of cardiovascular and metabolic abnormalities [30]. Moreover, hypovitaminosis D can relate to cardiac hypertrophy and heart failure [30]. Vitamin D deficiency leads to the development of arterial hypertension, cardiac hypertrophy, and atherosclerosis in animal model [31]. Vitamin D is a novel endocrine regulator of the renin-angiotensin system, as low vitamin D can overstimulate the renin-angiotensin system which causes cardiovascular injuries and HF [32]. Calcium associated with vitamin D also plays a role in reducing the risk of CVD [33]. Also, inadequate amount of calcium availability can negatively affect the intracellular signaling and smooth muscle contractions in the chambers of the heart which can lead to heart failure and cardiovascular diseases [15].

The intake of calcium supplements alone is still controversial as some studies showed that increasing calcium intake will cause an elevation in blood calcium level and the chance of calcium deposition in the lumen of the blood vessels causing their narrowing and stiffness [34]. Optimal bone and cardiovascular system healthiness requires intaking both dietaries, calcium and vitamin D, together.

Certain limitations of this study should be noted. Information regarding the physical activity and the possibility that the recruited females were on systemic estrogen use was missing from patient files. Estrogen is playing an important role in keeping the bones strong and healthy; thus, estrogen deficiency can be related to the onset of osteopenia in both sexes and increases the CVD susceptibility.

## Conclusion

Elderly Arab of both sexes suffering from osteopenia is at higher risk to develop CVD that can lead to HF. Moreover, a low level of daily vitamin D and calcium could protect from the incidence of future CVD in patients suffering from low BMD. The association between high calcium intake and CVD risk needs to be assessed further in additional studies.

## Data Availability

The data that support the findings in this study are available from the corresponding author upon reasonable request.
